# Applications of event-related potentials in Alzheimer’s disease: a systematic review and analysis

**DOI:** 10.3389/fnagi.2025.1513049

**Published:** 2025-05-02

**Authors:** Qian Liang, Zhilin Chen, Xiaohui Tang, Xijin Wang

**Affiliations:** Department of Neurology, Tongji Hospital, School of Medicine, Tongji University, Shanghai, China

**Keywords:** Alzheimer’s disease, cognitive dysfunction, mild cognitive impairment, event-related potentials, diagnosis, prediction

## Abstract

The prevalence of Alzheimer’s disease (AD) has been increasing continuously, representing a major issue for public welfare. Detecting cognitive impairment due to AD at its early stages is an urgent prerequisite for therapeutic treatment to slow or halt disease progression before major brain damage occurs. Event-related potentials (ERPs) are a noninvasive neurophysiological technique with the advantages of objectivity, ease of operation, and real-time reflection of cognitive processing in the brain. The purpose of this paper is to comprehensively assess the application value of ERP in AD. By systematically searching relevant literature in PubMed, Web of Science and Scopus databases, and merging and analyzing the literature included in the study, we explored the roles of various components of ERP in the diagnosis, disease monitoring and pathological mechanism research of AD, and provided a comprehensive overview of the current status and prospect of the application of ERP in AD.

## 1 Introduction

AD is an age-related degenerative disease of the central nervous system characterized by progressive cognitive dysfunction and behavioral impairment (Soria et al., 2019; Alzheimer's Association, 2023). Memory impairment is a typical symptom of AD. In the early clinical stage, patients experience declarative memory impairment phenomena such as forgetting recent conversations, names, and times of familiar relatives, which significantly reduces their social and life ability (Knopman et al., 2021; Se et al., 2021; Kumar et al., 2024). As the disease progresses, patients will also have non-declarative memory impairment and emotional problems such as apathy, depression, irritability. In severe cases, personality and behavior changes may occur (Lane et al., 2018). The pathogenesis of AD remains unclear. The occurrence of AD is related to a variety of factors. *β*-amyloid deposition and neurofibrillary tangles caused by abnormal phosphorylation of Tau protein are two characteristic pathological changes in AD (Wegmann et al., 2021; Naseri et al., 2019; Rostagno, 2022; Scheltens et al., 2021). Current statistics show that dementia is one of the leading causes of disability in people over 65 years old worldwide (Jia et al., 2020), which affects 55 million people worldwide and is expected to more than triple by 2050 as the population ages, with prevalence increasing with age (Long, 2023). AD is the most common type of dementia in old age (Kumar et al., 2024; Breijyeh and Karaman, 2020), causing not only a serious economic burden to patients and their families but also great challenges to national medical systems and society, so it is very important to prevent the occurrence of AD and diagnose it in early clinical stage. ERPs are special brain-evoked potentials that represent potential activity associated with a certain stimulus extracted from scalp EEG using superimposed averaging methods (Randolph and Helfrich, 2019; McWeeny and Norton, 2020; Randolph and Helfrich, 2019; McWeeny and Norton, 2020). ERPs are used to record neural responses to specific sensory, cognitive, and motor events (Tsai and Liang, 2021), and widely applied in studying the pathophysiological mechanisms of disease like AD, Parkinson’s disease (PD), stroke, schizophrenia, etc. This article mainly summarizes the clinical application of ERPs in AD and studies its correlation with the progression of the disease through literature review and analysis.

## 2 Overview of ERPs

### 2.1 Development of ERPs

EEG is a method of recording brain activity using electrophysiological indicators, which is the basis for the generation of ERPs (Randolph and Helfrich, 2019; Donoghue and Voytek, 2022). In 1875, Richard Caton et al. recorded electrical activity on the surface of the exposed brain of rabbits, 1924 Hans Berger first recorded EEG on the scalp of patients with skull injuries and healthy people (Randolph and Helfrich, 2019; Berger, 1929). After the discovery of EEG, scientists tried to study evoked potentials (EP) using stimulation-induced brain waves. Sensory evoked potentials were first recorded on the scalp of the awake human brain by Pauline and Hallowell Davis in 1935–1936 (Davis et al., 1939). With the deepening of research, it was found that EP can be caused not only by external stimulation but also by active top-down psychological factors, so “stimulation” was changed to “event” and “evoked potential” was changed to “event-related potential” (Randolph and Helfrich, 2019). In 1962, Galambos and Sheatz published the first ERPs paper on computer-averaged superposition (Galambos and Sheatz, 1962); In 1964, Grey Walter et al. discovered the first cognitive ERPs component (contingent negative variation, CNV) (Walter et al., 1964), marking the beginning of a new era in ERPs research; In 1965, Sutton, Braren, Zubin, and John discovered the P300 (Sutton et al., 1965), more and more researchers began to explore the components of ERPs related to cognition.

### 2.2 The main components of ERPs

The components of ERPs are related to the brain’s information processing of stimuli, which can be divided into exogenous components related to the processing of physical stimuli and endogenous components related to mental processing. The endogenous components are mismatch negativity (MMN), P300 (Sutton et al., 1965), CNV, and N400 (Kutas and Hillyard, 1980), which reflect different higher cognitive processing processes. In addition, the components of ERPs also include movement-related potentials (BSP, MP, RAF), lateralized readiness potential (LRP), processing negativity (PN), recognition potential (RP), error-related negativity (ERN, Pe, FN), visual C1, P1, visual N2, etc. (Donoghue and Voytek, 2022; Sutton et al., 1965; McWeeny and Norton, 2020; Pratt and Kappenman, 2011; Pratt, 2011; Friedman et al., 2001; Kappenman et al., 2021). Table 1 summarizes the various ERP components and associated properties.

**Table 1:** The various ERP components and associated properties.

ERP component	Latency (ms)	Scalp distribution	Functions and related cognitive processes	Paradigm	Factor
N100	100–200	Widely distributed, with large frontal and central areas.	Early sensory processing of stimuli and attentional orientation. When an individual notices an external stimulus, the N1 amplitude increases, reflecting the brain’s initial screening and orientation response to the stimulus.	The visual or auditory odd-ball paradigm (presenting a series of frequent standard stimuli and occasional deviant stimuli) requires the participant to respond to the deviant stimulus.	Physical characteristics of the stimulus (e.g., intensity, frequency, etc.), state of attention, difficulty of the task.
P200	200–300	Central-parietal lobe region	It is related to the evaluation and classification of stimuli and may involve the processing of the initial meaning of the stimulus. In some tasks that require a simple classification of stimuli, the P2 amplitude varies depending on the class of stimulus.	Color classification task (presenting different colored shapes and asking the participant to respond to a specific color).	The type of stimulus, the individual’s expectations, and the relevance of the task.
N200	250–350	Frontal lobe, central area.	Associated with conflict monitoring, response inhibition, and working memory. For example, in the Stroop task, when the meaning of a color word is inconsistent with the actual color, a large N2 wave is triggered, reflecting the brain’s detection and processing of conflicting information.	Stroop task (presenting color words, the meaning of the word may or may not be consistent with the font color, asking the participant to report the font color), Go/No-Go task (presenting a stimulus, asking the participant to respond to a specific stimulus, inhibiting the response to other stimuli).	Degree of conflict, difficulty of response, working memory load.
P300	300–800	The parietal lobe is predominantly and widely distributed.	It is related to the integration of information, decision-making and memory updating. When an individual receives a meaningful, task-related stimulus, a distinct P3 wave occurs. P3 amplitude is related to the probability of stimuli, task difficulty, and the allocation of cognitive resources to individuals.	In the classic ODD-ball paradigm, the participant is required to count or respond otherwise to a rare target stimulus.	The probability of the stimulus (rare stimuli elicit greater P3), the importance of the task, the level of arousal of the individual.
N400	300–500	Widely distributed, with the largest in the middle of the left temporal lobe	It is mainly related to semantic processing, especially the semantic understanding of words and sentences. When semantically abnormal or incontextual words are presented, a large N400 wave is thrown.	Semantic priming task (presenting a primer word followed by a target word, asking participants to judge whether the target word is related to the primer word), sentence comprehension task (presenting a series of sentences containing sentences with abnormal semantics).	Semantic relevance, contextual consistency, lexical familiarity.
Late Positive Potential (LPP)	500	The central-parietal region is also found in the frontal lobe.	Associated with emotional processing, motivation, and evaluation. When viewing emotional images (e.g., pleasant, unpleasant, or neutral images), the LPP amplitude increases under emotional picture conditions, reflecting the brain’s ongoing processing and evaluation of emotional stimuli.	Emotional picture viewing task (presenting pictures of different emotional categories, requiring participants to watch and evaluate their emotions), reward task (giving different levels of reward feedback after completing the task).	The emotional valence of the stimulus (pleasant or unpleasant), the size of the reward, the emotional state of the individual.
MMN	100–250	Forehead- central area, usually larger on the left side.	Automatic detection of changes in stimuli without the need for attentional involvement. It occurs when there is a deviation stimulus that deviates from the standard stimulus in the auditory stimulation sequence, reflecting the brain’s automatic comparison and recognition mechanism of sensory information.	In the passive auditory ODD-ball paradigm, the participant does not need to respond to the stimulus, but simply listens quietly to a sequence of standard stimuli and deviation stimuli.	The type and degree of stimulus change, the probability of stimulus presentation, and the participant’s arousal state.
N170	170	The occipital-temporal lobe region, more pronounced in the right hemisphere.	Early specific processing of visual stimuli, such as faces, is thought to be associated with the early stages of face recognition.	Face recognition task that presents a variety of face pictures, including normal faces, inverted faces, or other object pictures.	Visual characteristics of stimuli, familiarity of faces, distribution of attention.
P50	50	Widely distributed, the frontal lobe and central zone are relatively distinct.	It plays a role in the sensory gating process, participates in the screening and filtering of early sensory information, and reduces the interference of irrelevant information.	The bipulse auditory stimulation paradigm, which presents two auditory stimuli with short intervals, measures the response to the second stimulus.	Stimulus interval, individual attention state, nervous system excitability.

ERPs reflect the integrated cognitive functions of the brain, including the processes of information extraction, processing, and output, etc. The greatest advantage of ERPs in neuroscience is its high temporal resolution, which can accurately and without delay respond to the brain’s activity at each moment in time, and they are widely used in the assessment of cognitive impairment in various central nervous system (CNS) diseases as well as in the diagnosis of psychiatric and psychological disorders.

## 3 Methods

### 3.1 Search strategy

This systematic review followed the Preferred Reporting Items for Systematic Reviews and Meta-Analysis (PRISMA) flow diagram.^1^

**Figure 1:**
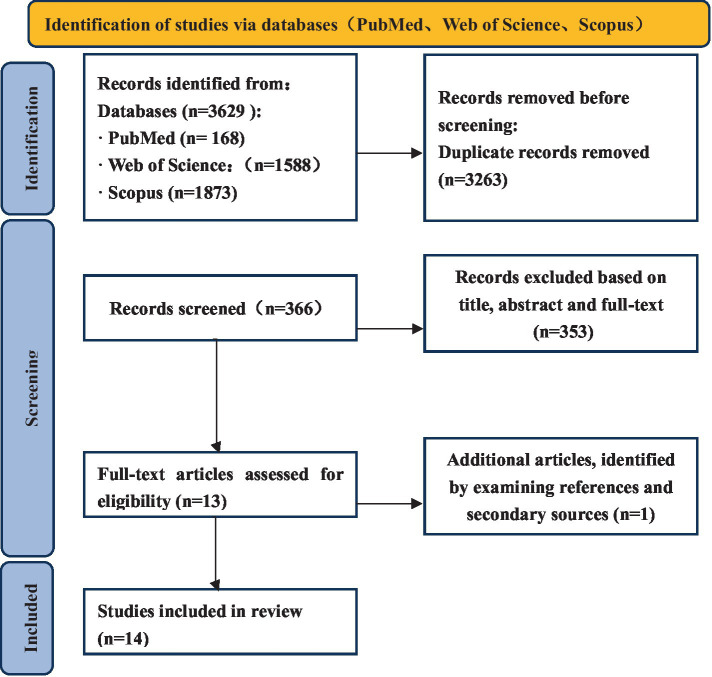
PRISMA diagram for systematic literature reviews.

The PRISMA flow diagram is presented in Figure 1.

A literature search in PubMed, Web of Science and SCOPUS was performed in March 2024 using the following search string: PubMed: (“Alzheimer Disease”[Mesh]) OR (“Cognitive Dysfunction”[Mesh] OR “Dementia”[Mesh]) AND (“Event-Related Potentials, P300”[Mesh]); Web of Science: TS = (“Alzheimer Disease” OR “Alzheimer’s disease” OR “Cognitive Dysfunction” OR “AD” OR “Dementia”) AND TS = (“Event - Related Potentials” OR “event - related potential” OR “ERP”); Scopus: TITLE - ABS - KEY(“Alzheimer Disease” OR “Alzheimer’s disease” OR “Cognitive Dysfunction” OR “AD” OR “Dementia”) AND TITLE - ABS - KEY(“Event - Related Potentials” OR “event - related potential” OR “ERP”). The search was limited to human participants and publications in English. All articles published before March 2025 were included. All search results were aggregated in Excel for Windows; duplicates were discarded, so unique references were retained at first.

### 3.2 Study selection

Records were screened according to title and abstracts by two independent researchers. Relevant articles and studies in which eligibility could not be determined based on title or abstract were selected for full-text review. Disagreement between the reviewers was resolved through debate, and the resulting decisions were unanimous.

Studies were eligible for inclusion only if: (I) Participants with AD were diagnosed by formal criteria (McKhann et al., 1984; McKhann et al., 2011); (II) MCI participants were diagnosed according to the Petersen criteria, including only the amnestic types (aMCI) (Petersen et al., 1999); (III) The control group was healthy individuals matched for age and gender; (IV) Used ERPs to detect the relevant indicators, and data on the amplitude and latency of the ERP components (e.g., P300, N400, etc.) were explicitly reported.

Studies with the following conditions were rejected for inclusion: (I) Duplicate publications; (II) Literature for which full text is not available and for which key data are missing; (III) Non-original research literature such as reviews, commentaries, and conference abstracts; (IV) Literature on subjects with other serious neurological or psychiatric disorders that may interfere with ERP results.

Finally, we checked the reference list of included articles, and the references cited within these sources to supplement our database searches, ensuring a comprehensive capture of relevant literature.

### 3.3 Quality evaluation

The Newcastle-Ottawa Scale (NOS) (Higgins et al., 2011; Wells et al., 2013) was used to assess the quality of the included case–control studies. The scale is scored from three dimensions: the selection of study subjects, comparability between groups, and the measurement of exposure factors, with a full score of 9, and a score of ≥7 is considered high-quality research.

## 4 Results

The search produced 3,629 articles, of which 168 were from PubMed, 1,588 were from Web of Science and 1,873 were from Scopus. 3,263 duplicate articles were removed. After the title, abstracts and full-text screening had been completed, 353 articles were removed. Finally, 13 articles were identified as meeting the inclusion criteria. Subsequently, our comprehensive search strategy, which included examining the references of these articles and their secondary sources, led to the identification of 1 more studies, culminating in a total of 14 relevant articles. 2 out of 14 studies employed a longitudinal design, whereas a cross-sectional approach was used in the remaining studies. Table 2 summarizes the details of all included studies, including first author, year of publication, sample size (experimental and control), study design, main results, etc.

**Table 2:** ERPs studies focusing on AD.

First author and year	Participants	Study design	ERP component	Main results	Sensitivity/Specificity
Fruehwirt et al. (2019)	63 subjects (31 with possible, 32 with probable AD diagnosis)	Longitudinal (18 months)	P300, N200, P50	P300 and N200 latency significantly correlated with disease severity in probable AD, whereas P50 amplitude did not. P300 latency, which showed the highest correlation coefficients with MMSE.	n.a.
Bennys et al. (2007)	30 AD, 20 MCI and 10 control subjects.	Cross-sectional	P300, N200	ERPs were significantly different between the groups (AD, MCI, control subjects), with a marked increase of P3 latencies. N200 latencies strongly discriminated MCI from control subjects.	P3 latencies: se 87–95%/ sp. 90–95% N2 latencies: se 70–75%/ sp. 70–90%
Cecchi et al. (2015)	103 subjects with probable mild AD and 101 healthy controls.	Cross-sectional	P3b, N200	P3b amplitude was typically smaller, and P3b latency was longer in subjects with AD. N200 latencies longer.	n.a
Sabbaghi et al. (2021)	75 mild AD patients (age: 76.2 ± 0.74) and 95 age-matched HCs (age: 73.2 ± 0.71).	Cross-sectional	auditory ERPs	Using extracted features and a radial basis function neural network, a high overall diagnostic accuracy of 98.3% was achieved.	High
Chapman et al. (2011)	43 elderly individuals diagnosed with MIC.	Longitudinal	ERPs	Supported by a cross-validation where the prediction accuracy was 70–78%, features the posterior probability for each individual as a method of determining the likelihood of progression to AD.	High
Eyamu et al. (2023)	160 MCI and 407 cognitively normal individuals.	Cross-sectional	ERPs	Multiple logistic regression analyses confirmed the association between some ERP and behavioral measures with MCI prevalence.	n.a
Quiroz et al. (2011)	21 familial AD: 10 presymptomatic subjects positive for the PSEN1 mutation (carriers) and 11 siblings without the mutation.	Cross-sectional	ERPs	The ERP measures to predict the presence of AD pathology.	se 72.7%/ sp. 81.8%
Zappoli et al. (1995)	24 early AD and 10 age-matched healthy volunteers.	Cross-sectional	CVN	Compared with age-matched healthy individuals, there were significant differences in CNV in early AD patients.	High
Tsolaki et al. (2017)	21 AD, 21 MCI and 21 healthy elderly.	Cross-sectional	MMN, P300	Compared with healthy people, the amplitude of MMN and P300 in AD patients is reduced and the latency is prolonged.	High
Chrysa and Papadaniil (2016)	21 AD, 21 MCI and 21 healthy volunteers.	Cross-sectional	MMN, P300	The amplitude of MMN and P300, and statistically significant prolonged latencies, correlated with dementia progression.	High
Bobes et al. (2010)	24 pre-carriers, 25 symptomatic carriers of the E280A (*PS-1*) and 27 noncarriers (same families).	Cross-sectional	N400	The symptomatic-carrier group performed worse in the matching task and had lower N400 amplitudes than both asymptomatic groups.	High
Gmehlin et al. (2011)	20 young subjects (age 26 ± 5 years), 23 elderly subjects (age 72 ± 5 years)	Cross-sectional	P30, P50, P200, N100	Both age groups exhibited clear inhibition in preattentive P50 and attention-modulated (N100) components, whereas P30 was not attenuated.	n.a
Lazarou et al. (2018)	12 HC, 14 SCI, 17 MCI and 14 AD participants.	Cross-sectional	N170	The amplitude of N170 elicited after negative facial stimuli could be modulated by the decline related to pathological cognitive aging and can contribute in distinguishing HC from SCI, MCI, and AD.	n.a
Fide et al. (2019)	24 AD and 23 healthy elderly.	Cross-sectional	P100, N170	Increased P100 amplitude and latency, decreased N170 amplitude were observed in AD compared to controls.	High

### 4.1 The response time of CVN in AD patients is prolonged

CNV is one of the earliest reported cognitive ERPs, which can detect the arousal level and sustained attention of people under stress, and CNV is a negative potential deflection between the warning stimulus (S1) and the target stimulus (S2), mainly distributed in the frontal region. In the typical CNV task paradigm, S1 and S2 appear in pairs, 30 pairs or more at a time, the subjects need to respond as soon as possible after S2, and the stimulation can be visual, auditory or audio-visual (de Tommaso et al., 2020). The study found that compared with age-matched healthy individuals, there were significant differences in CNV in early AD patients, especially in the late CNV before S2, which may suggest that CNV can provide clues for researching the pathophysiological changes of early AD and may be an important means to identify early AD (Zappoli et al., 1995; Zappoli et al., 1991).

### 4.2 The amplitude of MMN in AD patients is reduced and the latency is prolonged

MMN is produced when deviant stimuli are randomly inserted into a series of repeated standard stimuli, reflecting auto-processing of brain to deviant stimuli (Garrido et al., 2009). It originates from the temporal lobe primary and frontal lobe secondary auditory cortex (Sams et al., 1985) with a latency of 100-250 ms. The study found that AD patients have defects in selective attention and working memory capacity, patients are easily distracted by irrelevant information, have a reduced range of memory, decay rapidly, also have executive dysfunction; compared with healthy people, the amplitude of MMN in AD patients is reduced (Tsolaki et al., 2017) and the latency is prolonged (Horvath et al., 2018), reflecting the degree of cognitive impairment in AD higher than in healthy older adults (Chrysa and Papadaniil, 2016; Chrysa and Papadaniil, 2016), indicating that AD patients have impaired attention and reduced auto-processing ability and MMN may be able to predict and identify potential risk groups for AD.

### 4.3 The amplitude of P300 in AD patients is reduced and the latency is prolonged

P300 is the third positive wave of ERPs, and its discovery is due to the improvement of the average superposition of neural electrophysiological signals (Friedman et al., 2001). In 1965 Sutton first described the P300: a late positive component that peaks at about 300 ms (Sutton et al., 1965), it consists of three subcomponents, including P3a, P3b, and nP3 (Friedman et al., 2001; Polich and Criado, 2006; Polich, 2007). P300 originates in different regions of the brain, including the temporoparietal junction, the medial temporal lobe complex, and the lateral prefrontal cortex (Huang et al., 2015; Kugler et al., 1993; Briand et al., 2007), P3a reflects frontal function (Polich, 2004), and P3b is associated with the temporoparietal pathway (Huang et al., 2015; Polich, 2004; Warren et al., 2023; Halgren et al., 1980; Fruehwirt et al., 2019). Task-induced P300 processes involve attention, memory, comparison, and decision-making, which constitute the advanced functions of information processing (Hansch et al., 1982). The P300 usually varies with the task intensity and the decision complexity, and its amplitude is also regulated by various factors, such as stimulus significance and resource availability (Chrysa and Papadaniil, 2016). The study found that the P300 latency period prolonged and the amplitude decreased in AD patients compared with healthy people (Duncan et al., 2009; Bobes et al., 2010; Bennys et al., 2007), and the prolongation of the latency period is highly correlated with the severity of cognitive impairment, the more severe the cognitive impairment, the longer the latency period, especially in language, memory, and executive ability (Kostova et al., 2014). Therefore, P300 plays an important role in the evaluation and diagnosis of AD.

### 4.4 The amplitude of N400 in AD patients is reduced and the latency is prolonged

The typical N400 task paradigm uses semantically congruent and semantically incongruent stimuli to detect language function. For example, the semantic priming task is the appearance of semantically relevant or irrelevant target stimuli after stimulation with meaningful cues (words, pictures, etc.) (Duncan et al., 2009). When selecting lexical stimulus materials, factors such as word frequency and concreteness need to be considered. The ratio between semantically congruent and semantically incongruent stimuli is often 1:1, and the number of semantically incongruent stimulus trials is ≥40. The amplitude of the typical N400 task paradigm is related to semantic conflict, the stronger the semantic conflict, the larger the amplitude of the evoked N400 (Bobes et al., 2010). It has been found that semantically conflicting paired words can also induce the N400, and the amplitude also becomes smaller as the frequency of stimulus repetition increases (Kostova et al., 2014). The presence of deficits in language and symbol integration processes is one of the typical clinical symptomatic features of AD (Mueller et al., 2018), so N400 may be an indicator of language-related processing deficits in AD. Semantic priming and repetition priming are more frequently used in the study of semantic memory function in AD (Joyal et al., 2020; Perri et al., 2019), and semantic incongruence will cause a larger N400 (Kara and Federmeier, 2005). Compared with healthy older adults, the N400 amplitude of AD patients is smaller, and AD patients have a longer delay of N400 in semantic processing tasks, suggesting that there is a deficit in semantic priming and semantic processing in AD patients (Joyal et al., 2020).

### 4.5 The latency of N100 and P200 in AD patients is prolonged

The N100 and P200 are exogenous components related to sensory processing, peaking at approximately 100 ms and 200 ms post-stimulation, respectively. They are associated with attention and their amplitude and latency change with age (Bennys et al., 2007). In auditory tasks involving memory, both the N100 and P200 show increased latency with age (Gmehlin et al., 2011). Research has demonstrated that compared to healthy elderly individuals, AD patients exhibit prolonged latencies of N100 and P200 (Bennys et al., 2007), particularly in response to visual stimulation (Yamasaki et al., 2012; Cecchi et al., 2015). The abnormality of ERPs components in AD patients indicates early-stage impairment of sensory function. These abnormalities reflect differences in cognitive processing between AD patients and healthy elderly individuals, serving as neuroelectrophysiological biomarkers for distinguishing between the two groups.

### 4.6 The amplitude of N170 in AD patients is decreased

N170 is a classical visual ERPs component associated with face processing, which amplitude peaks between 140 and 200 ms after face stimulation. N170 is mainly involved in the perceptual process of facial or object feature integration, such as the recognition of human facial identity information (Feuerriegel et al., 2015). One of the most typical clinical symptoms of AD is forgetting familiar faces of family members and companions. This symptom is often attributed to underlying memory impairment (Mazzi et al., 2020), recent studies have found that there are also defects in visual processing (Yamasaki et al., 2012; Fernández et al., 2018; Yamasaki et al., 2012; Fernández et al., 2018; Yamasaki et al., 2012; Fernández et al., 2018). Studies have shown that AD patients have difficulties in color and depth perception, visuospatial organization, control of visual attention, and visual search (Lavallee et al., 2016). The amplitude of N170 evoked by processing facial visual stimuli is reduced in AD patients compared with healthy elderly people, which also suggests that N170 can effectively distinguish healthy elderly people from AD patients, thereby reducing the incidence of AD (Lazarou et al., 2018; Lin et al., 2019). Some studies have found that in the passive observation task using emotional facial expressions, the N170 amplitude of AD patients is significantly lower than that of healthy elderly people (Fide et al., 2019), reflecting the impairment of emotion recognition in AD patients, and impaired emotion recognition may be an early sign of cognitive processing impairment (Fujie et al., 2008). In addition, when only the eyes, nose, mouth and facial contour of the face were presented, the N170 amplitude of the AD patients was also reduced, reflecting the impairment of the ability of perceptual organization during face encoding processing in AD patients (Saavedra et al., 2012). Combined with previous studies, AD patients have not only memory impairment, but also higher-level visual perceptual processing defects, that is, the perception ability of human facial stimulation is decreased, which also suggests that future studies need to use electrophysiological indicators to better predict and find the high-risk groups of AD in the population, so as to reduce the incidence and number of AD (Sabbaghi et al., 2021; Chapman et al., 2011; Eyamu et al., 2023; Quiroz et al., 2011; Polikar et al., 2007; Sabbaghi et al., 2021; Chapman et al., 2011; Eyamu et al., 2023; Quiroz et al., 2011; Polikar et al., 2007).

## 5 Conclusion and prospects

### 5.1 Potential clinical implications of ERPs in the diagnosis of AD

ERPs have been utilized in the evaluation of cognitive processes, particularly in the early diagnosis of AD and mild cognitive impairment (MCI) (Bennys et al., 2007; Cecchi et al., 2015; Sabbaghi et al., 2021; Vecchio and Määttä, 2011). Studies have demonstrated the potential of ERPs as non-invasive, objective, and low-cost biomarkers for the early detection of AD patients, with high diagnostic accuracy achieved using specific ERP features and classifiers (Sabbaghi et al., 2021; Babiloni et al., 2020). ERPs have been shown to provide powerful and innovative tools for early AD diagnosis, reflecting differences in brain electrophysiology underlying cognitive functions in brain disorders such as dementia and MCI (Eyamu et al., 2023; Bennys et al., 2017).

As a neurodegenerative disease with unknown pathogenesis, the early differential diagnosis of AD is very important. The ERPs component reflects the neurodegenerative process of AD, and its sensitivity holds great promise for detecting and quantifying the presymptomatic stages of AD. Studies have found that AD patients have varying degrees of delay and amplitude changes in each component of ERPs. Early exogenous component changes are the manifestation of sensory impairment, which may be used to distinguish healthy elderly from AD patients. The late endogenous component changes can help to assess the changes in cognitive processes in patients, and the abnormalities of these components can help to differentiate and diagnose the disease. For example, the decrease of N170 amplitude reflects the defect of visual perceptual processing; The prolonged latency of MMN and P300 can be used to detect attention disorders, which is beneficial to the early diagnosis and evaluation of patients; The decrease and delay of N400 amplitude can be used to evaluate language impairment and memory impairment, and track the development of the disease. These components can not only distinguish the healthy elderly from the patients, but also be used to identify the different stages of AD. Therefore, linking ERPs to the diagnosis of the disease could facilitate AD assessment and diagnosis in clinical practice.

### 5.2 Limitations of current ERPs in AD research

ERPs have shown promise in the study of AD, but they also face several limitations. Understanding these limitations is crucial for advancing the field and improving the utility of ERPs in AD research. ERPs signals are usually weak and can be easily affected by background noise. This noise may come from the environment, equipment, or the patient’s own physiological activity (e.g., muscle activity, eye movements, etc.). In order to improve the signal-to-noise ratio, complex data processing and filtering are required, which can affect the accuracy and reliability of the data. ERPs research often requires the design of specific cognitive tasks whose complexity and difficulty may affect patient performance and ERPs response. Different tasks may elicit different ERPs components, and there are large differences in ERPs responses between different individuals, which makes it difficult to draw consistent conclusions in group studies. In addition, factors such as age and gender can also affect ERPs responses, adding to the complexity of data interpretation. While ERPs have high temporal resolution, may not capture very brief neural activity changes in some cases. At the same time, ERPs have low spatial resolution, making it difficult to precisely localize brain activity.

### 5.3 Future prospects of ERPs in AD research

As technology and research methods continue to evolve, ERPs is expected to play a greater role in early diagnosis, disease monitoring, treatment effect evaluation and pathological mechanism research. In the future, higher resolution ERPs equipment can be developed to capture and analyze brain electrical activity more precisely. Using artificial intelligence and machine learning technology, develop more advanced signal processing and filtering algorithms to improve the quality and accuracy of ERPs signals and analysis. Developing multimodal data analysis methods that integrate and interpret data from different technologies to provide a more comprehensive approach to diagnosis and monitoring. ERPs can also be combined with other neuroimaging technologies (e.g., MRI, PET) and biomarkers (e.g., blood and cerebrospinal fluid markers) to gain a deeper understanding of the pathological mechanisms of AD and provide more comprehensive pathological information, thereby providing new ideas and methods for new treatments. At the same time, developing an ERPs-based early warning system to help identify high-risk groups and intervene before symptoms appear. Designing more rigorous longitudinal studies to dynamically monitor changes in cognitive function and assess disease progression, and developing individualized treatment and rehabilitation plans for patients.

Future research should focus on the integration of ERPs with other biomarkers, such as protein biomarkers measured in the cerebrospinal fluid, to improve the differential diagnosis of AD in MCI patients and enhance the predictive models for AD likelihood. There is a need for further investigation into the potential of ERPs, particularly the P300 and N200 components, as preclinical markers of AD, aiming at the earliest possible diagnosis of the disease (Polikar et al., 2007; Bennys et al., 2017). In summary, the meta-analyses and studies on ERPs in Alzheimer’s disease have demonstrated the potential of ERPs, particularly the P300 and N200 components, as sensitive and reliable measures of cognitive deficits associated with early AD. While there are promising applications of ERPs in the early detection of AD, further research is needed to address the limitations and to explore the integration of ERPs with other biomarkers for improved diagnostic accuracy and predictive models.

## Footnotes

^1^ https://www.bmj.com/content/339/bmj.b2700
